# Metabolism of *β*-valine via a CoA-dependent ammonia lyase pathway

**DOI:** 10.1007/s00253-015-6551-z

**Published:** 2015-05-26

**Authors:** Marleen Otzen, Ciprian G. Crismaru, Christiaan P. Postema, Hein J. Wijma, Matthew M. Heberling, Wiktor Szymanski, Stefaan de Wildeman, Dick B. Janssen

**Affiliations:** Department of Biochemistry, Groningen Biomolecular Sciences and Biotechnology Institute, University of Groningen, Nijenborgh 4, 9747 AG Groningen, The Netherlands; DSM Pharmaceutical Products, Geleen, The Netherlands; BioBased Materials, Faculty of Humanities and Sciences, Maastricht University, Chemelot, The Netherlands

**Keywords:** *β*-valine, CoA ligase, Ammonia lyase, BvaA, BvaB1, BvaB2

## Abstract

*Pseudomonas* species strain SBV1 can rapidly grow on medium containing *β*-valine as a sole nitrogen source. The tertiary amine feature of *β*-valine prevents direct deamination reactions catalyzed by aminotransferases, amino acid dehydrogenases, and amino acid oxidases. However, lyase- or aminomutase-mediated conversions would be possible. To identify enzymes involved in the degradation of *β*-valine, a *Ps*SBV1 gene library was prepared and used to complement the *β*-valine growth deficiency of a closely related *Pseudomonas* strain. This resulted in the identification of a gene encoding *β*-valinyl-coenzyme A ligase (BvaA) and two genes encoding *β*-valinyl-CoA ammonia lyases (BvaB1 and BvaB2). The BvaA protein demonstrated high sequence identity to several known phenylacetate CoA ligases. Purified BvaA enzyme did not convert phenyl acetic acid but was able to activate *β*-valine in an adenosine triphosphate (ATP)- and CoA-dependent manner. The substrate range of the enzyme appears to be narrow, converting only *β*-valine and to a lesser extent, 3-aminobutyrate and *β*-alanine. Characterization of BvaB1 and BvaB2 revealed that both enzymes were able to deaminate *β*-valinyl-CoA to produce 3-methylcrotonyl-CoA, a common intermediate in the leucine degradation pathway. Interestingly, BvaB1 and BvaB2 demonstrated no significant sequence identity to known CoA-dependent ammonia lyases, suggesting they belong to a new family of enzymes. BLAST searches revealed that BvaB1 and BvaB2 show high sequence identity to each other and to several enoyl-CoA hydratases, a class of enzymes that catalyze a similar reaction with water instead of amine as the leaving group.

**Electronic supplementary material:**

The online version of this article (doi:10.1007/s00253-015-6551-z) contains supplementary material, which is available to authorized users.

## Introduction

Whereas the microbial metabolism of *α*-amino acids is well known, the bioconversion of *β-*amino acids leaves a wide spectrum of metabolic exploration. More insight into the enzymology of *β*-amino acid metabolism is desirable since it can lead to the discovery of enzymes that can be used in applied biocatalysis or incorporated in biosynthetic pathways. In this study, we aimed to understand the deamination mechanism of *β*-valine. For *α*-valine and other *α*-amino acids, several conversion types are described in the literature (Fernández and Zúñiga 2006; Massey et al. 1976). The most common enzymes in aliphatic amino acid (de)amination reactions include aminotransferases, amino acid dehydrogenases, and amino acid oxidases. Although these enzymes use different mechanisms for substrate deamination, all form a keto-product. Substrates such as *β-*valine that carry the amino group on a tertiary carbon atom cannot be deaminated by these enzymes due to the lack of a carbon-hydrogen bond. Consequently, it was expected that unusual deamination mechanisms could operate on these substrates.

The first step in the deamination of *β*-valine can be performed by an aminomutase, resulting in an *α-*amino acid that is deaminated by one of the common reactions mentioned above. Aminomutases catalyze a reversible intra-molecular amino group migration between vicinal carbon atoms. These enzymes are involved in processes such as the anaerobic biodegradation of lysine and the biosynthesis of several biologically active secondary metabolites, such as antibiotics (Walker et al. 2004; Yin et al. 2003). Aminomutase reactions proceed with the direct involvement of several cofactors, such as *S*-adenosylmethionine (SAM), [4Fe-4S] clusters, pyridoxal-5′-phosphate (PLP), coenzyme B_12_, or 4-methylideneimidazole-5-one (MIO) (Heberling et al. 2013; Wu et al. 2011; Frey and Reed 2011).

Deamination of *β*-valine can possibly also occur by a lyase reaction, with formation of the corresponding enoate. These reactions are well known for amino acid deamination; the most common examples being aspartase, methylaspartate ammonia lyase, and MIO-dependent ammonia lyases. Although these enzymes are structurally and mechanistically quite different, they all are restricted to substrates containing a carboxylate group in combination with an aromatic group (histidine, phenylalanine, and tyrosine ammonia lyases) or a second carboxylate (aspartase and aspartate ammonia lyase). These structural commonalities are explained by the need to delocalize negative charge to a carboxylate or aromatic functionality that is *beta*-positioned with respect to the carbon from which the amino group is eliminated. So far, these lyase reactions are not known to occur directly on amino acids that carry only a single carboxylate (such as valine or leucine) or *β*-amino acids (such as 3-aminobutyrate or *β*-alanine) (Bartsch et al. 2013).

Another possibility is that *β*-valine deamination is dependent on activation by conversion to a coenzyme A (CoA)-thioester, as described for the metabolism of *β*-alanine and *α*-lysine (Herrmann et al. 2005; Kreimeyer et al. 2007). *β*-Alanine is a naturally occurring *β*-amino acid found in vitamin B_5_ (pantothenic acid). In the metabolic degradation pathway, *β*-alanine is first activated by CoA in a reaction catalyzed by *β*-alanyl-CoA-transferase. Ammonia is then eliminated in a reaction catalyzed by *β*-alanyl-CoA/ammonia lyase (Herrmann et al. 2005). Additionally, anaerobic metabolism of *α*-lysine proceeds via several reactions yielding 5-amino-3-oxohexanoate. This reacts with acetyl-CoA resulting in acetoacetate and 3-aminobutyryl-CoA, which is successively deaminated to crotonyl-CoA by 3-aminobutyryl-CoA deaminase (Kreimeyer et al. 2007).

In this study, the deamination of *β-*valine was studied in detail using the newly isolated *β-*valine-degrading microorganism *Pseudomonas* sp. strain SBV1, to potentially discover a novel pathway for *β*-amino acid deamination. This study revealed that metabolism of *β*-valine is dependent on the *β*-valinyl-CoA ligase BvaA and the two novel CoA-dependent ammonia lyases BvaB1 and BvaB2.

## Materials and methods

### Strains and chemicals

*Escherichia coli* TOP10, MC1061, and C41(DE3) were purchased from Invitrogen. *Pseudomonas fluorescens* Pf0-1 was kindly provided by Prof. M.W. Silby (University of Massachusetts Dartmouth, USA) and *P. fluorescens* Pf-5 was obtained from American Type Culture Collection (ATCC). The *β-*valine degrading bacterium SBV1 (DSMZ, accession number DSM 29543) was isolated from residential grass soil (The Netherlands), by growth selection on YCB medium containing *β-*valine as a sole nitrogen source.

Adenosine monophosphate (AMP), adenosine diphosphate (ADP), adenosine triphosphate (ATP), CoA (trilithium salt), and isopropyl-β-d-thiogalactoside (IPTG) were obtained from Sigma. *β-*Valine was purchased from Fluorochem (Germany). dl-*α*-valine and *α*-ketoglutarate (disodium salt) were purchased from Fluka. The protease inhibitor cocktail complete (EDTA-free) was bought from Roche. Restriction enzymes were purchased from New England BioLabs and used according to the manufacturer’s protocol.

### Cultivation conditions

*E. coli* cells were grown at 37 °C in LB medium (Sambrook et al. 2001) or M9 medium (Sigma) containing 2 mM MgSO_4_, 0.4 % glucose, 0.1 mM CaCl_2_, 0.02 % thiamine, and 1 % casamino acids. *P. fluorescens* cells were grown at 30 °C on LB, nutrient broth (NB; Sigma), or nitrogen-free minimal medium (MM) (Gabor et al. 2004), supplemented with citrate (10 mM) or glucose (0.2 %) as carbon source and (NH_4_)SO_4_ or *β-*valine (5–10 mM) as nitrogen source. When required, leucine (5 μg/mL) and ampicillin (50 μg/mL) or tetracycline (12.5 μg/mL) were added to the media. For the preparation of agar plates, the medium was supplemented with 2 % agar or 1.5 % agarose, rinsed with H_2_O.

### Plasmid construction

Primers are listed in Table S1. The pBADNK plasmid is a pBAD/MycHisA-derived expression vector (Invitrogen) in which the *Nde*I site is removed and the *Nco*I site is replaced by *Nde*I.

For the purification of BvaA, plasmid pBad-bvaA-his was constructed containing an in-frame fusion of the *bvaA* gene to a Myc*-*His-tag, behind the *araBAD* promoter region. For this purpose, the *bvaA* gene was amplified from plasmid p4-D1 (described in this paper) and primers Lig_fw and Lig_rev. The resulting product, lacking the stop codon of the *bvaA* gene, was then digested with *Nde*I and *Xho*I, and ligated into *Nde*I-*Xho*I-digested pBADNK.

For the purification of BvaB1 and BvaB2, plasmids pET-bvaB1-his and pET-bvaB2-his were constructed containing an in-frame fusion of either the *bvaB1* or the *bvaB2* gene to a His-tag behind the T7 promoter. To this purpose, the *bvaB1* and the *bvaB2* genes were amplified using plasmid p4-D1 and either primers LyS2_fw and LyS2_rev or LyS3_fw and LyS3_rev. The resulting products, lacking the stop codon of the genes, was then digested with *Nde*I and *Xho*I, and ligated into *Nde*I-*Xho*I-digested pET21a (Agilent).

### Cosmid library generation and screening

Genomic DNA of SBV1 cells was partially digested with *Sau*3AI to an average fragment size of 20–30 kb. Fragments were ligated into the *Bam*HI-linearized cosmid cloning vector pLAFR3 (Friedman et al. 1982). Packaging was done in phage particles using the Gigapack III-XL kit according to the instructions of the manufacturer (Agilent). Phage particles were transfected to *E. coli* TOP10 cells, and clones were selected on LB agar with tetracycline.

Triparental mating (Ditta et al. 1980) was carried out by replica plating donor cells on LB-agar plates treated with an exponentially growing culture of the helper strain *E. coli* HB101(pRK600) (Kessler et al. 1992). After overnight incubation, these plates were replica plated onto NB-agar plates treated with an overnight-grown NB culture of the recipient strain *P. fluorescens* Pf0-1. The plates were incubated overnight at 30 °C, after which transconjugants were selected by replica-plating onto MM agarose plates containing 5 mM sodium citrate, 10 mM ammonium sulfate, and tetracycline.

To select potential *P. fluorescens* Pf0-1 clones that were complemented for their growth deficiency, all transconjugants were replica-plated to agarose plates containing 5 mM citrate and 10 mM *β-*valine as sole carbon and nitrogen sources. Positive clones were identified based on the ability to form a clearly visible colony on this medium. To test whether identified positive clones were able to produce ammonia from substrate, transconjugants were tested by an ammonia release assay (Berthelot assay, Fawcett and Scott 1960). For this purpose, a small amount of cell material was transferred from a fresh NB plate to a microtiter plate (MTP) well containing 150 μl of 5 mM *β*-valine in 20 mM KPi buffer (pH 7.5). The MTP was covered with BreathSeal (GE Healthcare) and incubated overnight in a tabletop MTP shaker at 30 °C. The cells were centrifuged and to 20 μl of supernatant, 30 μl of 0.025 % phenol (vol/vol) in 0.3 N NaOH, 30 μl of 0.01 % sodium nitroprusside, and 30 μl of 20 mM NaOCl were added. This mixture was incubated for 15 min at 30 °C. Production of free ammonia by *P. fluorescens* Pf0-1 clones was detected by the appearance of a blue color.

### DNA sequencing

For the identification of strain SBV1, the 16S ribosomal RNA (rRNA) gene was amplified using primers 27F and 1492R (Table S1). The resulting product was subsequently purified using the PCR Purification Kit (Qiagen) and sequenced by GATC Biotech. The nucleotide sequence was deposited at GenBank (accession number KM657826). Subsequent identification was performed online, using the EzTaxon database (Chun et al. 2007).

In order to obtain a partial sequence of the SBV1 genome, total genomic DNA from SBV1 cells grown on MM containing 10 mM *β*-valine and 20 mM glucose was isolated according to a described procedure (Poelarends et al. 1998). The resulting genomic DNA was subjected to paired-end sequencing by Baseclear BV. The genome sequencing was done using an Illumina (Solexa) GA*IIx* Genome Analyzer. Sequencing yielded 7.2 million reads of ~50 bp. These were assembled using the CLC Genomics Workbench software, resulting in 996 contigs with a total length of 6,440,520 bp.

Sequencing of pLAFR3 inserts from specific *P. fluorescens* Pf0-1 transconjugants was done at GATC Biotech, using various primers. The complete insert sequence of p4-D1 was assembled by combining the contigs of the partial genome sequence and primer walking (GATC Biotech), resulting in a sequence of 25,618 bp (GenBank accession number KM595283).

### Expression and purification

For expression of BvaA-His, an overnight culture of *E. coli* MC1061 containing pBAD-bvaA-his was 100-fold diluted in M9 medium and incubated at 37 °C in a rotary shaker. When the culture reached an OD_600_ of ~0.6, arabinose (0.02 %) was added to the medium and the culture was incubated for an additional 64 h at 17 °C.

For expression of BvaB1-His or BvaB2-His, an overnight culture of *E. coli* C41(DE3) containing pET-bvaB1-his or pET-bvaB2-his was 100 times diluted in LB medium and incubated at 37 °C in a rotary shaker. When the culture reached an OD_600_ of ~0.6, 0.5 mM IPTG was added to the medium and the culture was incubated for an additional 16 h at 30 °C.

For the purification of BvaA-His enzyme to 40–50 % purity and BvaB1-His or BvaB2-His enzyme to more than 90 % purity, cells were harvested by centrifugation (20 min, 6000 × *g*, 4 °C). Subsequently, cells were resuspended in buffer I (50 mM 4-(2-hydroxyethyl)-1-piperazineethanesulfonic acid (HEPES), 500 mM NaCl, 20 mM imidazole, pH 8, protease inhibitor cocktail) and lysed by sonication. To remove unbroken cells and cell debris, lysates were centrifuged at 30,000 × *g* for 45 min. The extracts were separated on a 5-ml Hi-trap (GE Healthcare) column connected to an Äkta purifier, and the proteins were eluted using increasing concentrations of elution buffer (50 mM HEPES, 500 mM NaCl, 50 mM imidazole, pH 8). The imidazole was removed using an EconoPac 10DG desalting column (Bio-Rad) against buffer II (20 mM HEPES, 150 mM NaCl, 10 % glycerol, pH 8). Enzyme concentrations were determined using Bradford reagent.

### Enzyme activity assays

CoA ligase activity towards *β*-valine was determined by following the substrate-dependent formation of AMP by reverse-phase high-performance liquid chromatography (HPLC). Standard reaction mixtures contained 50 mM HEPES (pH 8), 150 mM NaCl, 5 mM MgCl_2_, 5 mM ATP, 2.5 mM CoA, 2.5 mM *β*-valine, and 0.7 mg/ml BvaA-His in a total volume of 500 μl. Reactions were carried out at 30 °C. Samples were taken and quenched by addition of 0.8 % formic acid. Levels of AMP, ADP, ATP, CoA and *β*-valinyl-CoA were determined by HPLC using a Gemini C18 column (5 μm particle size, 250 × 4.6 mm), connected to a Jasco UV-2075 detector set at 254 nm. Compounds were eluted using buffer A (25 mM KH_2_PO_4_, 2.5 % triethylamine, set to pH 6.5 using H_3_PO_4_, and 5 % methanol) with a gradually increasing concentration of elution buffer B (buffer A with 50 % methanol; 0–2 min, 0 %; 2–20 min, 0–46 %; 20–30 min, 46–100 %; 30–32 min, 100 %; 32–35 min, 100–0 %; 35–45 min, 0 % of eluent B). Typical retention times were 10 min for AMP, 12 min for ADP, 13 min for ATP, 23 min for *β*-valinyl-CoA, 24 min for CoA, and 36 min for methyl-crotonyl-CoA.

Lyase activity was determined by monitoring enzyme-specific depletion of *β*-valinyl-CoA. Since no pure CoA-adduct was available, the level of *β*-valinyl-CoA was calculated based on the assumption that during the CoA ligase reaction, 1 mM substrate-dependent AMP release corresponds to 1 mM of *β*-valinyl-CoA product. Additionally, the level of methylcrotonyl-CoA was calculated based on the assumption that during the ammonia lyase reaction the substrate-dependent reduction of 1 mM *β*-valinyl-CoA corresponds to the formation of 1 mM of methyl-crotonyl-CoA product. For the analyses, *β*-valinyl-CoA was first produced in a reaction mixture containing 50 mM HEPES (pH 8), 150 mM NaCl, 5 mM MgCl_2_, 5 mM ATP, 2.5 mM CoA, 2.5 mM *β*-valine, and 0.7 mg/ml BvaA-His, in a total volume of 500 μl. After an incubation of 16 h at 30 °C, the samples were filtered to remove all ligase using Amicon Ultra-0.5 ml centrifugal filters (10 K, Merck Millipore). Subsequently, 0.025 mg/ml BvaB1-His or BvaB2-His was added to the samples. After different time intervals, enzymatic conversions in the samples were quenched by the addition of 0.8 % formic acid and samples were analyzed by reverse-HPLC analysis as described above.

### HPLC-MS

Identification of *β*-valinyl-CoA and methyl-crotonyl-CoA was performed in a LCQ Fleet Ion Trap mass spectrometer (Thermo Scientific). Reaction components were separated by HPLC using an Alltech Altima HP C18 column (3 μm, 100 × 2.1 mm). The CoA adducts were eluted using eluent A (water, 0.1 % formic acid) with an elution gradient of eluent B (acetonitrile, 0.08 % formic acid; 0–2 min, 0–2 %; 2–20 min, 2–15 %; 20–32 min, 15–80 %; 32–37 min, 80 %; 37–38 min, 80 0 %; 38–48 min, 0 % eluent B). The samples were analyzed in a negative ion mode, and mass spectra were collected over a scan range of *m*/*z* 500–1000. Typical retention times were 13 min for *β*-valinyl-CoA (*m*/*z* 864; M–H) and 28 min for methyl-crotonyl-CoA (*m*/*z* 847; M–H).

### UPLC-MS

To explore the substrate scope of the CoA ligase BvaA, the formation of aminoacyl-CoAs was analyzed by an Acquity TQD mass spectrometer (Waters). Reaction mixtures containing 2.5 mM of different substrates and CoA ligase were prepared, as described before. Substrates used in this assay included: *β*-valine, *β*-alanine, *α*-valine, phenylacetic acid, *β*-phenylalanine, *β*-glutamic acid, *β*-lysine, d-phenylalanine, l-phenylalanine, d-tyrosine, l-tyrosine, l-alanine, l-leucine, isoleucine, l-lysine, acetate, hexanoic acid, heptanoic acid, octanoic acid, benzoic acid, 4-chlorobenzoate, 3-aminobutyrate, and 2-aminoisobutyrate. Samples were taken and quenched by addition of 0.8 % formic acid. Subsequently, separation of the reaction components was performed by UPLC using an Acquity UPLC HSS T3 Column (1.8 μm, 2.1 × 150 mm). The components were eluted using a linear gradient (eluent A; water, 0.1 % FA, eluent B; 100 acetonitrile, 0.1 % FA). The samples were analyzed in a negative ion mode, and mass spectra were collected over a scan range of *m*/*z* 600–1100.

### Homology modeling

For the construction of a homology model of BvaA, the Phyre on-line server was used (Kelley and Sternberg 2009). To construct homology models of BvaB1 and BvaB2, the YASARA software (www.yasara.org) was used. Phyre selected the PDB structure of the hypothetical acyl-CoA ligase 3QOV as template for BvaA. YASARA constructed models of BvaB1 and BvaB2 based on the structure of the rat liver enoyl hydratase that was complexed with the inhibitor acetoacetyl-CoA (PDB 1DUB, Engel et al. 1996). Homology modeling of BvaB1 and BvaB2 was performed with the substrate *β*-valinyl-CoA bound in the active site. Comparison of the structures and preparation of the figures was performed using Pymol software (www.pymol.org).

MD simulations were carried out with the enzyme complexed with a transition state-like structure of the *β*-valinyl-CoA substrate (BvaB1, BvaB2) or the 3-amino-butyryl-CoA substrate (rat liver enoyl hydratase). This approach allows direct monitoring of residues that interact with the transition state and thereby lower the energy barrier for reaching the transition state (Gao et al. 2006; Pan et al. 2005; Zheng et al. 2008). To enable MD simulation of a transition state-like structure, distance restraints have to be used to prevent the structure from collapsing to the substrate or product structures which are lower in energy (Gao et al. 2006; Pan et al. 2005; Zheng et al. 2008; Xue et al. 2011). Angles and dihedrals were not constrained because these were not expected to differ significantly between substrates, transition state, and product. Since no quantum-mechanical modeling was available, the distances were constrained to the average of the corresponding distances in the substrate and product states, which should be sufficiently accurate as the difference with quantum mechanical calculations will only be tenths of an Ångström (Å). The distance between the *β*-valinyl-CoA nitrogen atom of the amine and the electrophilic carbon atom was set to 2.36 Å, which is the average of the Van der Waals contact distance (3.25 Å) and the length of a C–N bond (1.47 Å). Since the catalytic mechanism of BvaB1 and BvaB2 is expected to be similar to that of enoyl hydratases (Engel et al. 1996), which catalyze a dehydration of hydroxyesters instead of deamination of amino acids, the conserved Glu144 (Bca1 numbering) contributes to catalysis by stabilizing negative charge developing on the nitrogen atom of the *β*-valinyl-CoA moiety via hydrogen bonding while Glu164 will donate a proton to the leaving group amine. Therefore, the distance between Glu144 OE1 and the β-Val NHD1 hydrogen atom was constrained at 2.0 Å, similar to the length of a strong H-bond between a lysine and a glutamate (Musafia et al. 1995), and the distance between OE1 of Glu164 and the β-Val NHD1 hydrogen atom was set to 1.5 Å, which is in between the length of a strong H-bond and the length of a covalent N–H-bond (1.0 Å). These distances were constrained with a force constant of 200 N m^−1^. For simulations of the transition state with water attacking, the corresponding distances were 2.0 Å for the H-bond between the N-terminal Glu144 side chain oxygen and the nearest hydrogen atom of the water; 1.48 Å for the H-bond with the C-terminal Glu164, and 2.32 Å for the distance between the leaving group oxygen atom and the carbon atom to which it is covalently attached.

To carry out the MD simulations, either the obtained homology model or the X-ray structure of an enoylhydratase (PDB accession 1DUB) was used to prepare structures with the CoA thioester substrates bound together with the nucleophiles, ammonia, or water. Molecular dynamics (MD) simulations were carried with a leap-frog propagation scheme and a Berendsen thermostat in a rectangular simulation cell (Berendsen et al. 1984). The simulation cell extended around the protein at least 7.5 Å on each side and was filled with explicit water molecules and sodium chloride counter ions to a concentration of 0.5 %. The boundary conditions were periodic, the employed force field was Yamber3, and long range electrostatics (>7.86 Å) were calculated with a particle Mesh Ewald algorithms using fourth degree B-spline functions (Krieger et al. 2004; Essmann et al. 1995). After an energy minimization of the simulation box, each transition state-enzyme complex was subjected to three MD simulations that started with different initial atom velocities to create independent trajectories. The protein was heated from 5 to 298 K in the first 30 ps of the MD trajectory. The MD simulations were allowed to continue for another 70 ps at 298 K and from the last 50 ps, snapshots were collected to create an average structure that was visually inspected. Additional details of the protocol to carry out the MD simulations were published previously (Wijma et al. 2014).

## Results

### Isolation of bacterial strains using *β*-valine as a sole nitrogen source

In order to identify enzymes involved in the deamination of *β*-amino acids, microorganisms were enriched from garden soil by growth selection on medium containing *β-*valine as a sole nitrogen source. This resulted in the isolation of a bacterium termed SBV1. To confirm growth of strain SBV1 with *β-*valine as the sole nitrogen source, cells were cultured on minimal media (MM) supplemented with 30 mM glucose and 10 mM *β*-valine as sole carbon and nitrogen sources, respectively. Subsequently, *β-*valine levels were analyzed during cell growth using HPLC with UV detection at 210 nm. This revealed that SBV1 cells are able to degrade *β*-valine with a maximum growth rate of ~0.2 h^−1^ (Fig. S1).

To identify strain SBV1, the 16S rRNA gene was sequenced. The resulting nucleotide sequence was deposited at GenBank (accession number KM657826). A BLAST search using the partial 16S rRNA gene sequence revealed that SBV1 is a *Pseudomonas* species, closely related to *Pseudomonas avellanae* (99.1 % identity) and *Pseudomonas mandelii* (98.9 % identity). The isolate was deposited in the German Collection of Microorganisms and Cell Cultures (DSMZ, accession number DSM 29543).

### Identification of a putative *β*-valine degradation gene cluster

For the identification of enzymes required for *β*-valine degradation in SBV1 cells, total protein extracts of SBV1 cells were analyzed for the presence of proteins with induced expression on medium supplemented with *β*-valine. SDS-PAGE analyses of protein extracts from *β*-valine-grown SBV1 cells and ammonium-grown SBV1 cells revealed no significant difference indicating low expression levels of inducible *β*-valine degradation genes (data not shown).

Next, a genetic approach was followed. Genomic DNA of SBV1 cells was isolated and a partial genome sequence was determined by paired-end sequencing. Using a BLAST search, it appeared that the SBV1 genome is very similar to that of *P. fluorescens* Pf-5 and *P. fluorescens* Pf0-1. Growth assays revealed that both of these strains were not able to grow on medium supplemented with *β-*valine as a sole nitrogen source within 3 weeks, suggesting that although these strains are very similar on the genetic level, they lack the enzymes for *β-*valine metabolism.

To identify genes that are necessary for *β-*valine degradation in SBV1 cells, a SBV1 genome library was constructed in *E. coli*. For this purpose, genomic DNA of SBV1 cells was sheared to produce fragments of 20–30 kb, which were subsequently ligated into the cosmid vector pLAFR3. Screening of 2300 clones of the cosmid library in *E. coli* did not result in the identification of a clone that was able to grow on solid medium supplemented with *β-*valine after 3 weeks of incubation at 30 °C. Subsequent screening of the cosmid library in *P. fluorescens* Pf0-1 on plates containing *β-*valine and citrate resulted in two clones that were able to grow on this medium, called p5-F12 and p4-D1. An ammonia release assay using whole cells revealed that both clones produced free ammonia during growth on *β*-valine as a sole nitrogen source.

**Fig. 1:**
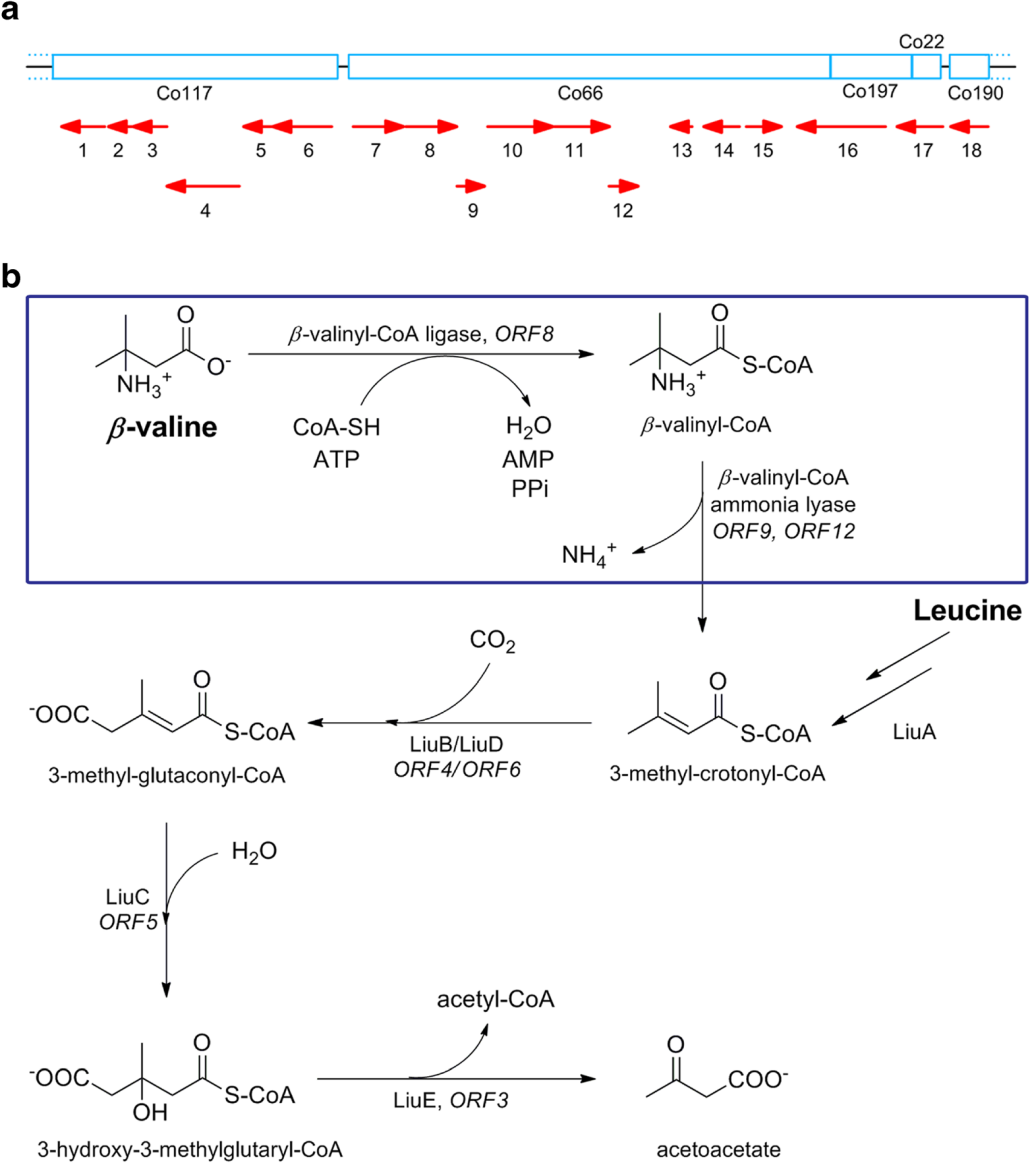
**a** Schematic representation of the organization of the ORFs identified in the 26,347 bp insert of plasmid p4-D1. **b** Proposed degradation pathway of *β*-valine by *Pseudomonas* sp. SBV1. The *boxed region* represents the putative initial steps in the *β*-valine degradation pathway, resulting in formation of 3-methyl-crotonyl-CoA, a common intermediate in the leucine degradation pathway, represented in the lower part. ORFs that are putatively important for *β*-valine degradation based on homology to enzymes with known function (Table 1) are numbered

Sequencing of the flanking regions from both inserts in combination with the available partial genomic information revealed that clones p5-F12 and p4-D1 contained a similar genomic fragment. The complete insert sequence of p4-D1 was assembled by combining the contigs of the partial genome sequence and primer walking, resulting in a sequence of 25,618 bp (Fig. 1a). The nucleotide sequence was deposited at GenBank (accession number KM595283).

Sequence analysis revealed the presence of 18 open reading frames (ORFs; Table 1; Fig. 1b). Interestingly, BLAST searches revealed that many ORFs encode CoA-dependent enzymes, among which several enzymes known to be involved in the leucine catabolic pathway. Based on this, a hypothetical pathway for *β-*valine metabolism in strain SBV1 was proposed, which partially overlaps with the leucine degradation pathway (Fig. 1b).

**Table 1:** List of ORFs identified in the 26-kb insert of p4-D1

ORF	Position in the insert (bp)	Enzyme	Seq. identity to known proteins (%, organism)	EC number	PDB ID
1	1429-245	Acetoacetyl-CoA thiolase	49, *Clostridium difficile*	2.3.1.16	4E1L
2	2204-1530	Gluathione *S*-transferase	28, *Pseudomonas aeruginosa*	2.5.1.18	4ECI
3	3132-2215	Hydroxymethylglutaryl-CoA lyase	75, *P. aeruginosa*	4.1.3.4	2FTP
4	5114-3129	3-methyl-crotonyl-CoA carboxylase	72, *P. aeruginosa*	6.4.1.4	3U9S
5	6024-5230	Putative enoyl-CoA hydratase	46, *Legionella pneumophila*	4.2.1.18	3I47
6	7644-6037	3-methylcrotonyl-CoA carboxylase	83, *P. aeruginosa*	6.4.1.4	3U9R
7	8228-9607	Amino acid transporter	23, *Escherichia coli*		3L1L
8 (BvaA)	9669-11069	Phenylacetate CoA ligase	36, *Burkholderia cenocepacia*	6.2.1.30	2Y4N
9 (BvaB1)	11069-11830	Enoyl-CoA hydratase	36, *Thermus thermophilus*	4.2.1.17	3HRX
10	11909-13690	Transcriptional regulator	46, *Vibrio cholerae*		4QHS
11	13735-15249	Acetyl-CoA acetyltransferase	30, *Mycobacterium smegmatis*	2.3.1.9	4EGV
12 (BvaB2)	15234-16034	Enoyl-CoA hydratase	48, *Mycobacterium avium*	4.2.1.17	3R0O
13	17509-16919	d-2-hydroxyacid dehydrogenase	48, *Klebsiella pneumoniae*	1.1.99.6	4 N18
14	18807-17827	Cre recombinase	25, *Enterobacteria* phage P1		1PVP
15	18989-19957	Putative integrase			
16	22807-20390	Hypothetical protein			
17	24375-23119	Hypothetical protein			
18	25616-24588	Hypothetical protein			

### Hypothetical *β*-valine degradation pathway

To enable growth on *β*-valine, uptake of *β*-valine may be facilitated by the transport protein encoded by ORF7. Inside the cell, *β-*valine may be activated by CoA in an ATP-dependent reaction catalyzed by a CoA-dependent ligase. Inspection of the insert for the presence of CoA ligase related sequences resulted in the identification of one ORF, ORF8. Database searches of the protein encoded by ORF8 revealed homology to the *Burkholderia cenocepacia* phenylacetate CoA ligase Paak1 (2Y4N, 35 % identity) and the *Pseudomonas putida* phenylacetate CoA Ligase (35 % identity). These enzymes catalyze the conversion of phenylacetic acid to phenylacetyl-CoA.

Subsequently, the *β-*valinyl-CoA adduct might be deaminated by an aminoacyl-CoA ammonia lyase, forming 3-methyl-crotonyl-CoA, a common intermediary metabolite in the leucine degradation pathway (Aguilar et al. 2006). No ammonia lyases that are active on *β*-aminoacyl-CoA have been described in the literature, with the exception of a *β-*alanine CoA ammonia lyase (Acl1 and Acl2; Herrmann et al. 2005) and 3-aminobutyryl-CoA lyase (Kreimeyer et al. 2007). Inspection of the insert for the presence of a homolog of this type of lyase did not result in the identification of such an ORF. However, further inspection of the sequenced region showed the presence of two ORFs (ORF9 and ORF12), which are highly similar (55 % DNA sequence identity) and homologous to genes encoding enzymes that belong to the family of enoyl CoA hydratases. Enoyl CoA hydratases catalyze the reversible hydration of the double bond of a 2-enoyl-CoA thioester, resulting in the formation of a *β*-hydroxyacyl-CoA thioester (Agnihotri and Liu 2003). Database searches with the protein sequence encoded by ORF9 revealed homology to *Metallosphaera sedula* 3-hydroxypropionyl-CoA dehydratase (34 % identity), an enzyme that converts 3-hydroxypropionyl-CoA to acryloyl-CoA (Teufel et al. 2009). Additionally, the protein revealed homology to the *Thermus thermophilus* PaaG (35 % identity), a predicted 2,3-dehydroadipyl-CoA hydratase of which the structure is solved (PDB 3HRX). Database searches of the protein encoded by ORF12 showed homology to *Mycobacterium avium* carnitinyl-CoA hydratase (3R0O; 48 % identity) and the *Proteus* sp. carnitinyl-CoA dehydratase (34 %), an enzyme that converts crotonobetainyl-CoA to l-carnitinyl-CoA (Engemann et al. 2001). Since some hydratases and ammonia lyases are structurally similar (e.g., fumarase and aspartate ammonia lyase; Puthan Veetil et al. 2012), it is conceivable that a hydratase-like protein encoded by one or both of these ORFs is involved in the deamination step.

Finally, the product 3-methyl-crotonyl-CoA can enter the leucine degradation pathway where it will undergo a carboxylation reaction to form to 3-methyl-glutaconyl-CoA. This reaction is catalyzed by 3-methyl-crotonyl-CoA carboxylase (ORF4, ORF6; Huang et al. 2011). In the next step, 3-methyl-glutaconyl-CoA can be hydrated by a hydratase (ORF5), forming 3-hydroxy-3-methylglutaryl-CoA. In the last step, the latter metabolite can be cleaved into acetyl-CoA and acetoacetate by hydroxymethylglutaryl-CoA lyase (ORF3).

### Characterization of BvaA

For the purification of the putative *β*-valinyl-CoA ligase encoded by *ORF8*, a C-terminal His_6_-tag version of this enzyme, named BvaA-His, was expressed in *E. coli*. A protein of the expected size was produced in low amounts, and most of it was present in the soluble fraction. Enzyme purification using Ni-NTA resin yielded ~2 mg purified enzyme/l of culture.

**Fig. 2:**
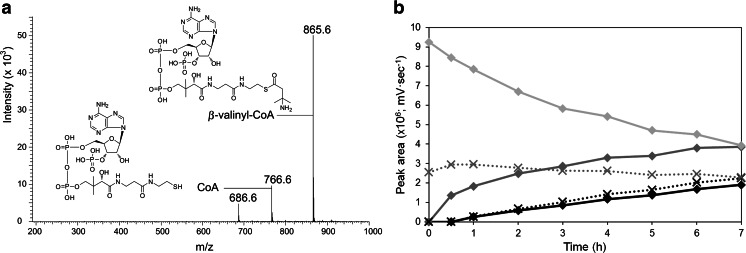
**a** MS spectrum of the single protonated form of *β*-valinyl-CoA (*m*/*z* of 866) and CoA (*m*/*z* of 767). **b** HPLC analyses monitoring the formation of AMP and *β*-valinyl-CoA by BvaA-His in time. *Lines*: , AMP; , ADP; , ATP. *Dotted lines*: , *β*-valinyl-CoA; , CoA

UPLC-MS analyses were performed to detect the formation of the *β*-valinyl-CoA adduct in a mixture containing ATP, CoA, *β*-valine, and BvaA-His. After 16 h of incubation at 30 °C, the presence of a parent ion of the expected size in a single protonated form was detected (Fig. 2a). Subsequently, levels of AMP, ADP, ATP, CoA, and *β*-valinyl-CoA were monitored in time by HPLC (Fig. 2b). This demonstrated that both the *β*-valinyl-CoA and the AMP levels increased in time. Calculation of the specific activity, based on substrate-dependent AMP production, revealed a rate of approximately 10 mU/mg.

**Fig. 3:**
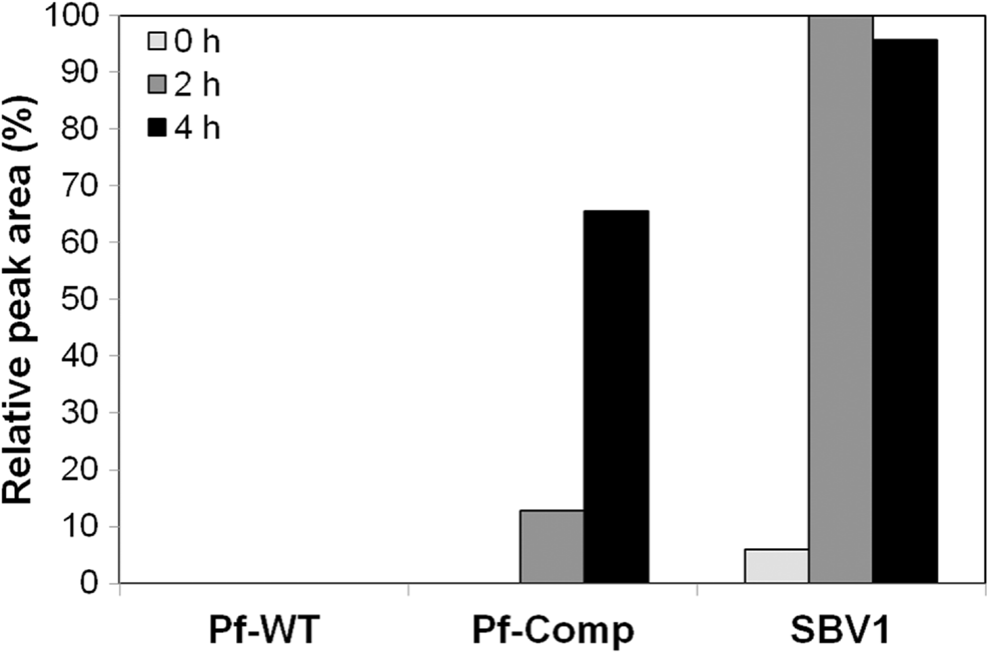
Conversion of *β*-valine to *β*-valinyl-CoA by cell-free extracts of cells grown on medium supplemented with *β*-valine and ammonium sulfate as nitrogen sources. Relative *β*-valinyl-CoA levels in the samples were analyzed after 0, 2, and 4 h of incubation using HPLC, where *β*-valinyl-CoA levels in SBV1 cell extracts after 2 h incubation was set at 100 %. *Pf-WT β*-valine growth-deficient strain *Pseudomonas fluorescens* Pf0-1, *Pf-Comp P. fluorescens* Pf0-1 containing the *bvaA* gene on the complementing plasmid p4-D1, *SBV1 β*-valine-degrading organism described in this study

To confirm that BvaA functions in *β*-valine activation, *β*-valinyl-CoA production was determined using protein extracts of various strains, including the *β*-valine growth-deficient strain *P. fluorescens* Pf0-1 (Pf-WT) and the *β*-valine-degrading organisms SBV1 and Pf0-1 containing the *bvaA* gene on the complementing plasmid p4-D1 (*Pf*-Comp). SBV1 protein extracts were used as a positive control. Cells were grown for 24 h on MM supplemented with a mixture of 2 mM ammonium sulfate and 5 mM *β*-valine as nitrogen sources. Subsequently, cell-free extracts of these cells were incubated in the presence of *β*-valine, CoA and ATP, and levels of produced *β*-valinyl-CoA were determined by HPLC (Fig. 3). This revealed that in cell-free extracts of *β*-valine-grown SBV1 cells already a low level of *β*-valinyl-CoA is present. After 2 h of incubation, *β*-valinyl-CoA levels were increased approximately 17-fold. Longer incubations did not increase the levels any further. Analyses of *β*-valinyl-CoA levels in samples containing protein extracts prepared from pf 0–1 cells revealed that *β*-valinyl-CoA is produced in time, but only in protein extracts prepared from cells containing plasmid p4D1 (Fig. 3). Taken together, this strongly implicates the *bvaA* gene in the activation of *β*-valine by ATP and CoA.

To determine the substrate range of BvaA-His, the ligase activity was tested using a wide range of substrates (see “Materials and methods”). Samples were analyzed by UPLC-MS for the production of an acyl-CoA. This revealed a minor activity with 3-aminobutyrate and *β*-alanine as possible substrates. However, adduct formation was much lower in comparison with the formation of *β*-valine-CoA. Note that CoA activation of the rest of the tested substrates, including several fatty acids and phenylacetic acid, was below the level of the detection. This suggests that BvaA is not a fatty acyl-CoA ligase or a phenylacetate CoA ligase.

### Characterization of BvaB1 and BvaB2

In order to analyze whether the putative *β*-valinyl-CoA ammonia lyases encoded by ORF9 and ORF12 are able to convert *β*-valinyl-CoA to 3-methyl-crotonyl-CoA, enzyme assays were performed. C-terminal His_6_-tag versions of both putative *β*-valinyl-CoA ammonia lyases, named BvaB1-His (ORF9) and BvaB2-His (ORF12), were expressed in *E. coli*. Proteins of the expected size were present in the soluble fraction. Enzyme purification using Ni-NTA resin resulted in 160 mg BvaB1-His and 14 mg BvaB2-His per liter of culture.

**Fig. 4:**
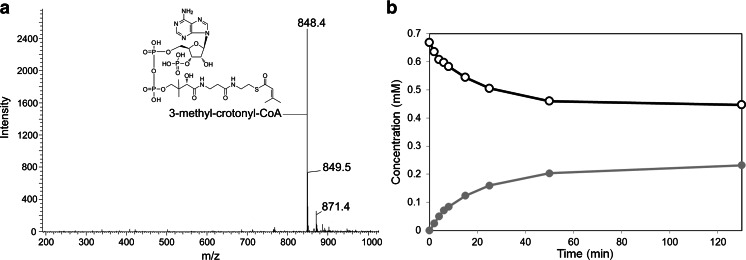
Identification of *β*-valinyl-CoA ammonia lyase product. **a** MS spectrum of the single protonated form of 3-methyl-crotonyl-CoA (*m*/*z* 849). **b** Conversion of *β*-valinyl-CoA (*open black symbols*) and formation of methyl-crotonyl-CoA (*closed grey symbols*) by purified BvaB1-his in time as monitored by HPLC

For the detection of the expected product 3-methyl-crotonyl-CoA, UPLC-MS analysis was performed on samples containing ATP, CoA, *β*-valine, BvaA-His, and either BvaB1-His or BvaB2-His. After 16 h of incubation at 30 °C, the presence of a parent ion with the expected size in a single protonated form was detected (Fig. 4a). This parent ion was observed in a mixture containing either BvaB1-His or BvaB2-His but not in a blank reaction without enzyme. These data reveal that both expected *β*-valinyl-CoA ammonia lyases are able to convert *β*-valinyl-CoA to 3-methyl-crotonyl-CoA.

To determine the specific activity of BvaB1-His and BvaB2-His, *β*-valinyl-CoA and 3-methyl-crotonyl-CoA levels were monitored in time by HPLC (Fig. 4b). This revealed that approximately 40 % of the available *β*-valinyl-CoA is converted to 3-methyl-crotonyl-CoA in the mixture. This degree of conversion was independent of the *β*-valinyl-CoA ammonia lyase used in the assay, suggesting an equilibrium. Calculation of the specific activity revealed similar rates for both lyases, approximately 1.5 U/mg for BvaB1-His and 0.9 U/mg for BvaB2-His.

## Discussion

This work investigated the *β*-valine degradation pathway in *Pseudomonas* species strain SBV1 with the overarching goal to obtain insight in the conversion of *β*-amino acids that cannot be deaminated by common aminotransferases, oxidase or dehydrogenase reactions. The results demonstrate that the key enzymes for the deamination of *β*-valine are a CoA-dependent ligase that activates *β*-valine and *β*-valinyl-CoA ammonia lyase that deaminates the product. These reactions result in the formation of free ammonia and 3-methyl-crotonyl-CoA, a common intermediate of the leucine degradation pathway (Massey et al. 1976).

For the identification of the genes involved in *β*-valine degradation by *P. fluorescens* SBV1, a closely related strain (*P. fluorescens* pf0-1) unable to grow on medium containing *β*-valine as a sole nitrogen source was used as a host for complementation of its *β*-valine growth deficiency with a cosmid gene library. This unveiled the identification of the *bvaA*, *bvaB1,* and *bvaB2*, genes that encode a CoA ligase (BvaA) and two CoA ammonia lyases (BvaB1 and BvaB2).

BLAST searches using the protein sequence of BvaA demonstrated sequence similarity to several phenylacetate CoA ligases, enzymes that catalyzes the conversion of phenylacetic acid to phenylacetyl-CoA (EC 6.2.1.30). In vitro characterization of BvaA revealed that this enzyme is indeed able to convert *β*-valine to *β*-valinyl-CoA in an ATP- and CoA-dependent manner. Interestingly, comparison of the biochemical properties of BvaA to different phenylacetate CoA ligases indicates a completely different substrate scope (Koetsier et al. 2009; Martinez-Blanco et al. 1990). The substrate range of BvaA is narrow, accepting only *β*-valine as a substrate and to a lesser extent 3-aminobutyrate. Phenylacetic acid, fatty acids, *α*-amino acids, and other *β*-amino acids such as *β*-lysine, were not converted. This observation suggests that BvaA does not functionally belong to the group of phenylacetyl-CoA ligases.

BvaA revealed a high sequence identity to the published *B. cenocepacia* phenylacetate CoA ligases, PaaK1 and PaaK2, which belong to the two-domain acyl-CoA ligases (Law and Boulanger 2011). Structural analyses of Paak1 and PaaK2 in complex with either ATP or phenylacetyl adenylate revealed several important residues for proper activity (PDB codes: 2Y27, 2Y4O, 2Y4N; Law and Boulanger 2011). Sequence alignments using the identified *β*-valinyl CoA ligase, PaaK1 and PaaK2 demonstrated that the ATP-binding pocket is present in the identified *β*-valinyl CoA ligase. To understand why the characterized *β*-valinyl-CoA ligase could not convert phenylacetic acid, a homology model of BvaA was inspected (see “Materials and methods”). Comparisons were made with 2Y4N, which is the crystal structure of PaaK1 in complex with phenylacetyl-CoA (Law and Boulanger 2011). The modeling indicates that the binding site is still present, but that the residues immediately lining the phenyl/*β*-valine binding site are partially altered. The following differences were observed (Y136 (BvaA) → L167 (Paak1), F141 → W172, A147 → D178, A214 → G245, I236 → N270, and Pro245 → Ile278). This large number of differences in residues that contact the phenyl ring in Paak1 (2Y4N) likely results in differences in shape and polarity of the substrate-binding site that explain why the phenyl substrate is not converted by the identified BvaA.

Characterization of the CoA-ammonia lyases suggest that both BvaB1 and BvaB2 belong to a new type of CoA-dependent ammonia lyases with some functional redundancy. Both enzymes are able to convert *β*-valinyl-CoA to 3-methylcrotonyl-CoA in vitro with a similar specific activity. Additionally, they display significant sequence homology to each other, but no remarkable similarity to other described CoA-dependent ammonia lyases, such as the *Clostridium propionicum β*-alanyl-CoA lyases (Herrmann et al. 2005).

**Fig. 5:**
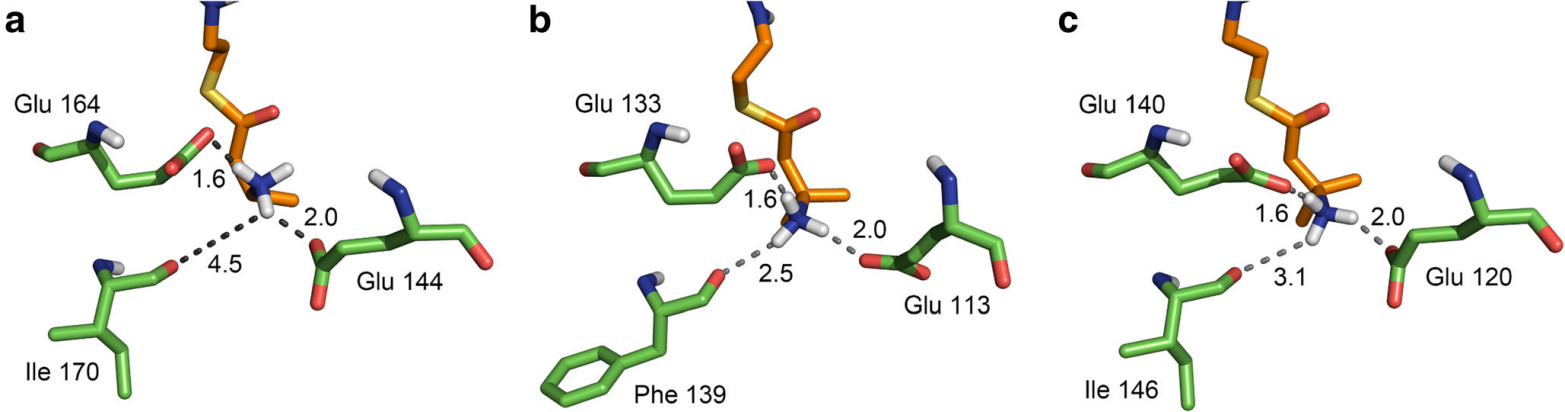
Active site geometries of (**a**) rat liver mitochondrial enoyl-CoA hydratase (PDB 1DUB) with modeled 3-amino-butyryl-CoA (*orange*) and BvaB1 **b** and BvaB2 **c** models with *β*-valinyl-CoA (*yellow*) docked. Only the aminoacyl part of the thioester of the substrate is shown, with the sulfur in *yellow* and the amino group to be eliminated in *blue*. The active-site residues potentially forming hydrogen bonds are indicated. Note that in 1DUB **a**, the distance for a third hydrogen bond is too Long. Distances in Ångström

BLAST searches using the protein sequences of BvaB1 and BvaB2 demonstrated high sequence identity to enoyl-CoA hydratases (EC 4.2.1.17), a class of enzymes that catalyze the reversible hydration of the double bond of a 2-enoyl-CoA thioester, resulting in the formation of a *β*-hydroxyacyl-CoA thioester (Agnihotri and Liu 2003). Homology models of BvaB1 and BvaB2 were created in order to rationalize why BvaB1 and BvaB2 are able to catalyze the deamination of *β*-valinyl-CoA, while the closely related enoyl hydratases perform a dehydration reaction. The models were based on the published crystal structure of the rat liver mitochondrial enoyl-CoA hydratase that was complexed with the inhibitor acetoacetyl-CoA (1DUB; Engel et al. 1996), an enoyl-CoA hydratase with homology to both BvaB1 (30 % identity) and BvaB2 (37 % identity). These homology models indicated that the active sites are highly similar (Fig. 5). Residues Glu144 and Glu164, which form hydrogen bonds with the hydroxyl group that is eliminated in enoyl hydratases, are conserved in BvaB1 and BvaB2 (BvaB1 E113 and E133; BvaB2 E120 and E140). Interestingly, the modeling of the active sites of the ammonia lyases predicts that they have an additional hydrogen bond acceptor, i.e., the backbone carbonyl oxygens of Phe139 and Ile146 in BvaB1 and BvaB2, respectively. This is in agreement with a hydroxyl group donating only one hydrogen bond and an uncharged amino group donating two hydrogen bonds. The MD simulations predict that the carbonyl oxygens of Phe139 and Ile146 indeed accept a hydrogen bond from the substrate amine group. This hydrogen bond is not present in the MD simulation of the rat enoyl-CoA hydratase with a modeled amine substrate, which might explain why BvaB1 and BvaB2 are CoA ammonia lyases instead of hydratases. The formation of the third hydrogen bond in the MD simulations of BvaB1 and BvaB2 seems to transpire from a change in the backbone of a loop region containing residues 168 to 177. The rat enoyl-CoA hydratase contains a proline at position 170, which is absent in BvaB1 and BvaB2, which may cause minor backbone changes that prevent formation of the third hydrogen bond in the rat enoyl-CoA hydratase.

A similar difference in H-bonding pattern appears to determine the substrate specificities of the well-studied enzymes aspartase (aspartate ammonia lyase) and fumarase (fumarate hydratase), which catalyze the addition of respectively ammonia or water to fumarate, resulting in aspartate or malate, respectively. In the literature it is described that also these enzymes are structurally very similar and share similar active site architecture (Puthan Veetil et al. 2012). Visual inspection of the solved X-ray structures (1FUO and 3R6V) show that, in the case of fumarase, a water molecule is tightly positioned with an unusually low B-factor (Weaver and Banaszak 1996). This water makes three H-bonds, twice as an H-bond donor (to Asn 141 and His 188) and once as an H-bond acceptor for Ser98. In aspartase (Fibriansah et al. 2011), the His and Asn are conserved but Ser98 has been replaced by an alanine, eliminating the only H-bond donor to the water. At the same time, in aspartase, Thr 101 can act as a H-bond acceptor for the ammonia, allowing its H-bonding potential to be fully satisfied while binding in a suitable orientation for catalysis to occur (data not shown).

## Electronic supplementary material

ESM 1 (PDF 79 kb)
