# Supplementary material for: Metabolism of β-valine via a CoA-dependent ammonia lyase pathway
Source: Appl Microbiol Biotechnol. 2015 May 26;99(21):8987–98. doi: 10.1007/s00253-015-6551-z (PMC4619459; doi:10.1007/s00253-015-6551-z)

Supplementary data

Applied Microbiology and Biotechnology

Metabolism of *β*-valine via a CoA-dependent ammonia lyase pathway

Marleen Otzen[1*], Ciprian G. Crismaru[1*], Christiaan P. Postema[1*], Hein J. Wijma[1], Matthew M. Heberling[1], Wiktor Szymanski[1], Stefaan De Wildeman[2,3] and Dick B. Janssen[1]

[*]These authors contributed equally to this work

1) Department of Biochemistry, Groningen Biomolecular Sciences and Biotechnology Institute, University of Groningen, The Netherlands
2) DSM Pharmaceutical Products, Geleen, The Netherlands
3) BioBased Materials, Faculty of Humanities and Sciences, Maastricht University, Chemelot, The Netherlands

Corresponding author:
Dick B. Janssen, Biotransformation and Biocatalysis, Groningen Biomolecular Sciences and Biotechnology Institute
University of Groningen, Nijenborgh 4, 9747 AG Groningen, The Netherlands
Tel. 0031-50-3634008, Fax 0031-50-3634165, e-mail: d.b.janssen@rug.nl.

Table S1 Primers used in this study

Primers	sequence
Lig_fw	5’ GAGTAGCATATGAAAATCTGCATGTGC 3’
Lig_rev	5’ CAGCTGCAGATCTCGAGTCCCGGCAATCCTCTCGATCAAGTTGG 3’
LyS2_fw	5’ GACGTACATATGAGCTTTGTATCGTATGAACG 3’
LyS2_rev	5’ GATGTCCTCGAGGCGTTTGTCGCGCCCAAACTG 3’
LyS3_fw	5’ GACACACATATGGACGGCAGAATAAAACAGTC 3’
LyS3_rev	5’ GCTTTACTCGAGGCGCCCAACCCAATTTGGTGTGC 3’
27F	5’AGAGTTTGATCMTGGCTCAG 3’
1492R	5’ GGYTACCTTGTTACGACTT 3’

Fig. S1 Conversion of *β*-valine by SBV1 cells. Cells were grown at 30°C in minimal medium containing 10 mM *β*-valine and 30 mM glucose; dotted lines - *β*-valine concentration; solid lines - optical density at 600 nm

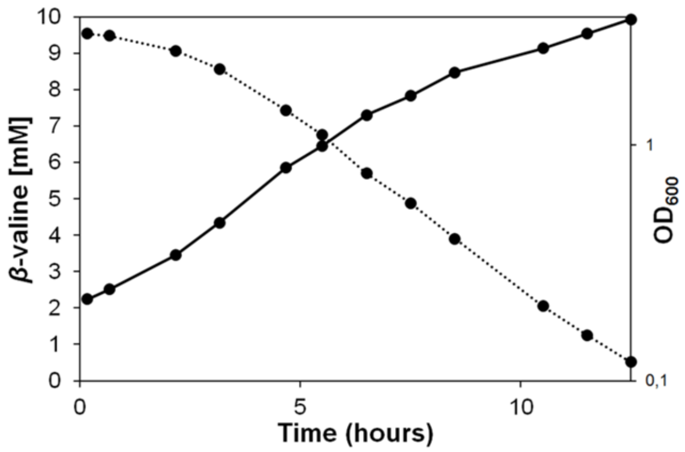

Supplement: Supplementary file 1 — (PDF 79 kb) [file 253_2015_6551_MOESM1_ESM.pdf]
